# Development and Validation of a Machine Learning Algorithm Using Clinical Pages to Predict Imminent Clinical Deterioration

**DOI:** 10.1007/s11606-023-08349-3

**Published:** 2023-08-01

**Authors:** Bryan D. Steitz, Allison B. McCoy, Thomas J. Reese, Siru Liu, Liza Weavind, Kipp Shipley, Elise Russo, Adam Wright

**Affiliations:** 1 Department of Biomedical Informatics, Vanderbilt University Medical Center, 2525 West End Ave., Suite 1475, Nashville, TN 37203 USA; 2 Department of Anesthesiology, Vanderbilt University Medical Center, 2525 West End Ave., Suite 1475, Nashville, TN 37203 USA

**Keywords:** clinical deterioration, early warning score, clinical informatics, machine learning.

## Abstract

**Background:**

Early detection of clinical deterioration among hospitalized patients is a clinical priority for patient safety and quality of care. Current automated approaches for identifying these patients perform poorly at identifying imminent events.

**Objective:**

Develop a machine learning algorithm using pager messages sent between clinical team members to predict imminent clinical deterioration.

**Design:**

We conducted a large observational study using long short-term memory machine learning models on the content and frequency of clinical pages.

**Participants:**

We included all hospitalizations between January 1, 2018 and December 31, 2020 at Vanderbilt University Medical Center that included at least one page message to physicians. Exclusion criteria included patients receiving palliative care, hospitalizations with a planned intensive care stay, and hospitalizations in the top 2% longest length of stay.

**Main Measures:**

Model classification performance to identify in-hospital cardiac arrest, transfer to intensive care, or Rapid Response activation in the next 3-, 6-, and 12-hours. We compared model performance against three common early warning scores: Modified Early Warning Score, National Early Warning Score, and the Epic Deterioration Index.

**Key Results:**

There were 87,783 patients (mean [SD] age 54.0 [18.8] years; 45,835 [52.2%] women) who experienced 136,778 hospitalizations. 6214 hospitalized patients experienced a deterioration event. The machine learning model accurately identified 62% of deterioration events within 3-hours prior to the event and 47% of events within 12-hours. Across each time horizon, the model surpassed performance of the best early warning score including area under the receiver operating characteristic curve at 6-hours (0.856 vs. 0.781), sensitivity at 6-hours (0.590 vs. 0.505), specificity at 6-hours (0.900 vs. 0.878), and F-score at 6-hours (0.291 vs. 0.220).

**Conclusions:**

Machine learning applied to the content and frequency of clinical pages improves prediction of imminent deterioration. Using clinical pages to monitor patient acuity supports improved detection of imminent deterioration without requiring changes to clinical workflow or nursing documentation.

**Supplementary Information:**

The online version contains supplementary material available at 10.1007/s11606-023-08349-3.

## INTRODUCTION

Unexpected clinical deterioration in hospitalized patients is a significant patient safety concern which can result in cardiac arrest, transfer to intensive care units (ICU), and preventable death.^1^ Patients who experience unanticipated deterioration often display signs of clinical instability in the preceding hours.^2–6^ Many organizations have adopted processes, such as Rapid Response Systems, to identify and intervene on patients likely to experience deterioration.^7, 8^ The efficacy of Rapid Response Systems is promising, with prior research highlighting a significant reduction in in-hospital mortality and cardiac arrest.^8–13^

Detecting patients at risk of clinical deterioration commonly relies on data contained within the electronic health record (EHR) to monitor physiological features. These early warning scores (EWS) use structured data, including patient demographics, vital signs, and nursing assessments to stratify patients by risk of deterioration.^5, 14–16^ EWS detect the sickest patients at risk of poor clinical outcomes, but often suffer from low discriminatory power and poor sensitivity to detect imminent events within the next 12-hours.^17–20^

Experienced clinicians accurately recognize clinical deterioration through intuition and knowledge about the patient before objective evidence is available.^21–23^ Incorporating features of clinical concern with structured EHR data can improve EWS performance.^21, 24^ Many rapid response systems incorporate intuition as a calling criteria for activation, which provides clinicians opportunity to request assistance at an early stage. However, barriers exist to calling on rapid response support, including lack of confidence and feelings of uncertainty often leads to delayed rapid response calls or escalation in care.^25^

Few EWS incorporate features that allow experts to include subjective assessments. Measures of worry are not directly captured in the EHR and mentions of concerns are often only documented in free-text notes or comments. Many healthcare institutions use pager messages, or brief unidirectional text-based messages from a healthcare worker to an individual’s pager, as an approach to indicate clinical needs and concerns. Healthcare workers communicate clinical concerns through electronic messages.^26^ Analysis of the content and frequency of pager messages to predict clinical outcomes represents a rich source of detail about a patient’s condition. We examined the efficacy of machine learning on pager messages sent by nurses to physicians to detect imminent clinical deterioration events in hospitalized patients.

## METHODS

We conducted this study at Vanderbilt University Medical Center (VUMC). VUMC is a large academic medical center located in middle Tennessee and provides referral care across the southeastern United States. VUMC includes an 864-bed adult hospital and sees nearly 2 million annual ambulatory visits. Clinicians at VUMC used an Epic EHR for all clinical functions. At VUMC, clinical pages are a primary mode of communication between healthcare workers. Most commonly, clinicians send pages about patients through the EHR by selecting the integrated care team paging activity. Clinicians can also send pages through a personnel and schedule management mobile phone application that is external to the EHR. Paging through the mobile application is used for administrative tasks, including to coordinate personnel and general bed management. Epic Secure Chat is not currently implemented at VUMC.

This study was approved and granted a waiver of consent by the Vanderbilt University Institutional Review Board. This study followed the Transparent Reporting of a Multivariable Prediction Model for Individual Prognosis or Diagnosis (TRIPOD) reporting guideline.

### Study Design and Population

In this retrospective study, we predicted clinical deterioration events among inpatients receiving care in hospital wards outside ICUs at VUMC. Our study included all patients who were at least 18 years old at the time of admission, and admitted between January 1, 2018 and December 31, 2020. We excluded patients receiving hospice or palliative care since these patients have different treatment plans and care processes. We labeled each encounter for the first of three deterioration events: Rapid Response activation, unplanned transfer to intensive care, and in-hospital cardiac arrest. The process for rapid response team (RRT) activation requires a hospital worker to call the VUMC emergency medical service (EMS) activation team in response to early warning criteria. EMS activation then calls a dedicated rapid response team to assess the patient and intervene as necessary. Unplanned transfers to intensive care included any transfer from a hospital ward to an ICU that did not include an intermediate surgery. In-hospital cardiac arrest was defined as any cardiac arrest on a hospital ward. All deterioration events were validated through retrospective chart review as part of ongoing rapid response quality improvement by an expert clinician (KS) on the VUMC RRT. Hospitalizations with a planned ICU stay or that did not include at least one sent page were excluded from our analysis. We also excluded encounters that were in the top 2% longest length of stay and did not experience a deterioration event as prior research has shown that excessively long hospitalizations are most caused by non-clinical factors.^27^

### Data Collection and Preprocessing

We collected data on all pages sent during our study. Page data included a unique identifier, page timestamp, patient name, medical record number (MRN), and message text. To ensure pages were sent about hospitalized patients, we matched pages to patient encounters by MRN and timestamp. If an MRN was not available, we mapped the page to an encounter using last name, timestamp, and room. 33% of pages could not be mapped and were excluded from our study. We manually reviewed a random subset of 250 unmatched pages and found that these discussed operating room availability, bed management, and personnel management. None of the pages included details about patient-specific care.

### Feature Selection

Model features included word embeddings, or numerical representations of the text from each page. We generated word embeddings using a clinical Bidirectional Encoder Representations from Transformers (BERT) model, which represents the content of clinical text.^28, 29^ Clinical BERT has been shown to develop meaningful representations of clinical text, including messages between healthcare team members.^26, 29^ To extract word embeddings from Clinical BERT, we first fine-tuned the model using the corpus of pages to ensures that the model accurately captures corpus-specific features. We used the fine-tuned model to process each page and extract word embedding from the last layer. We implemented Clinical BERT using the HuggingFace Transformers library.^30^

**Figure 1:**
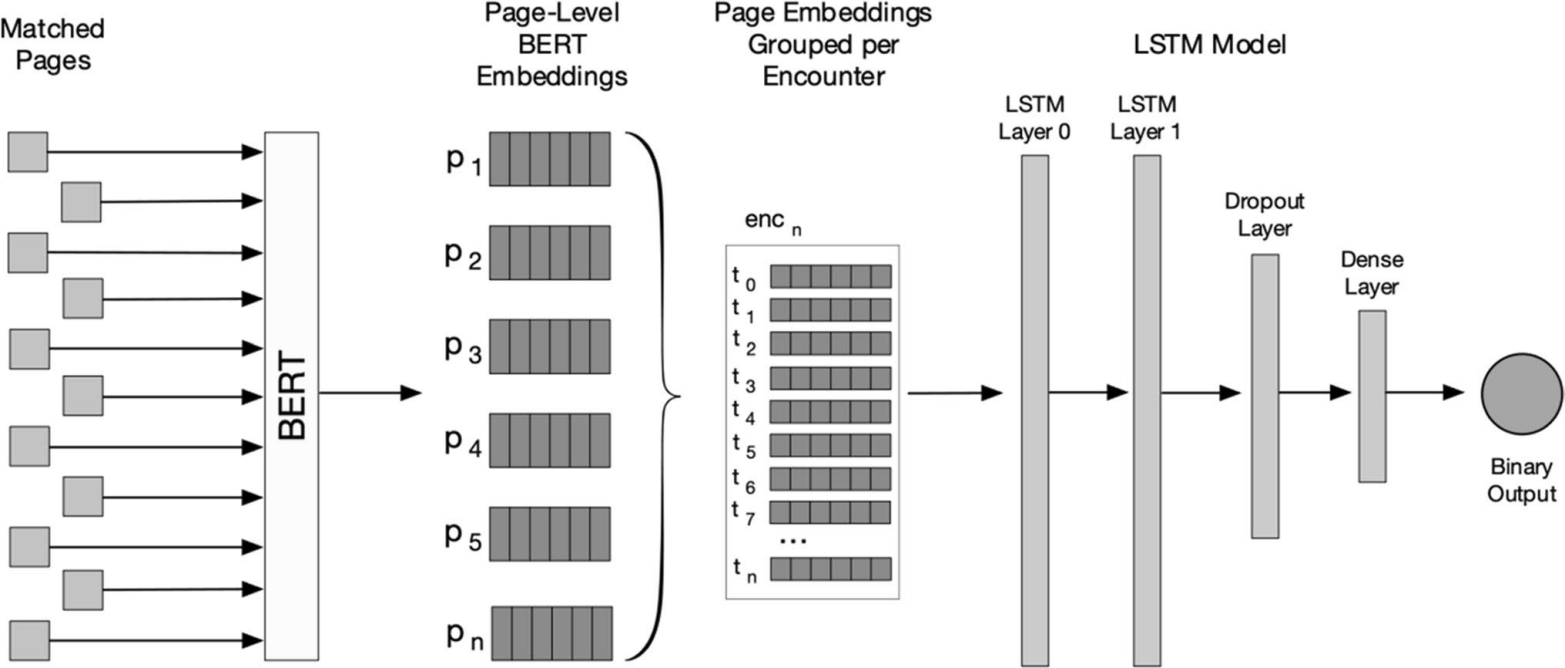
Page embedding and LSTM pipeline.

We combined page-level features into an encounter-level feature set for input into our models (Fig. 1). To create encounter-level features, we sequentially combined page-level features in ascending order. Encounter-level features contained only clinical pages sent during an encounter. We normalized timestamps into a single numeric value to represent the number of elapsed hours between hospital admission and time the page was sent. We created a washout period by truncating encounter-level features at a 3-, 6-, or 12-hour time horizon before the first deterioration event. All pages sent during an encounter, before the respective time horizon, were included in the feature set. We maintained all pages for hospitalizations that did not contain a deterioration event.

### Model Development and Evaluation

We trained two-layer long short-term memory (LSTM) machine learning models to predict clinical deterioration. Many machine learning models look at data at a single point in time. In contrast, LSTM models learn features and patterns from sequential data and are commonly used to make predictions over data in a timeseries, such as clinical pages during a hospital encounter.^31^ The feature embedding and LSTM model pipeline are presented in Fig. 1. We split the encounter-level data by year into training (70%) and testing (30%) datasets. We randomly split 20% of the training dataset to use during hyperparameter tuning and to enable early stopping during final model training to avoid overfitting. We tuned hyperparameters using random search with preset hyperparameter ranges (Appendix A). The testing dataset was held out from parameter tuning and used only to measure final results. We calculated validation loss after each iteration of hyperparameter tuning and model training to enable early stopping when validation loss plateaued or increased for three subsequent iterations. Following hyperparameter optimization, we developed models using the entire training dataset. We implemented our machine learning models and evaluated the performance using the Tensorflow (version 2.6.2)^32^ and scikit-learn (version 1.1.3)^33^ packages in Python 3.6.9.

### Statistical Analysis

We compared cohorts of patients who experienced deterioration events versus those who did not experience deterioration events using Welch t-tests for numerical features and Chi-square tests for categorical features. We considered a p value less than 0.05 to be statistically significant. Statistical analyses were performed using R version 4.1.2.

We measured classification model performance to predict clinical deterioration 3-, 6-, and 12-hours before the deterioration event using a held-out set of 30% of hospital encounters. We also compared classification performance stratified by type of deterioration event as a secondary analysis. Measured outcomes included area under the receiver-operating characteristic curve (AUROC), area under the precision-recall curve (AUPRC), sensitivity, specificity, F-score, and positive predictive value (PPV). We set thresholds for our models to achieve high sensitivity while maintaining acceptable PPV. Existing literature on EWS has targeted an acceptable PPV between 10% – 20%.^34–38^ As a sensitivity analysis, we reported classification metrics for every one-tenth change in predicted probability between 0 and 1.

We compared our model’s performance against recommended prediction thresholds from three common early warning scores: Epic Deterioration Index (EDI) [score ≥ 60],^39^ Modified Early Warning Score (MEWS) [score ≥ 5],^40^ and National Early Warning Score (NEWS) [score ≥ 7].^41^ Each EWS calculates a new score at regular thresholds. The EDI automatically calculates a score every 15 minutes; we re-calculated MEWS and NEWS scores each time a parameter was newly documented. We calculated encounter-level performance for each EWS. For encounters in which a deterioration event occurred, we obtained the highest EWS score in the time window from the start of hospitalization until k hours before a deterioration event. If a deterioration event did not occur, we obtained the maximum EWS score from the entire hospitalization. We indicated a predicted deterioration event when the maximum EWS score surpassed the prediction threshold. We also evaluated performance of EWS scores calculated in the k hours immediately before a deterioration event. We include this analysis across all deterioration events (Appendix B) and stratified by type of event (Appendix D).

## RESULTS

There were 87,783 patients (mean [SD] age at hospital encounter, 54.0 [18.8] years; 45,835 women [52.2%]) who experienced 136,778 hospitalizations and were the subject of 1,869,928 pages (mean [SD] 12.8 [13.9] pages per encounter). Deterioration events were recorded in 6214 encounters (4.5%). Rapid Response activations were the most common deterioration event (4121 [66.3%]), followed by ICU transfers (1753 [28.2%]) and in-hospital cardiac arrest (340 [5.5%]). Deterioration events occurred a mean [SD] 3.9 [3.9] days into the hospitalization. Table 1 details encounter-level population statistics.

**Table 1:** Encounter Population Statistics

	Encounters, No. (%)			
	Deterioration Event (*n* = 6,214)	No Deterioration Event (*n* = 130,564)	All Encounters (*n* = 136,778)	p-value^1^
Age (Years)				<0.001
Mean (SD)	60.3 (16.7)	53.7 (18.8)	54.0 (18.8)	
Median (IQR)	63 (22)	56 (30)	56 (31)	
Sex				
Male	3,356 (54.0)	63,400 (48.6)	66,756 (48.8)	<0.001
Female	2,858 (46.0)	67,150 (51.4)	70,008 (51.2)	
Unknown	0 (0.0)	14 (0.0)	14 (0.0)	
Race				0.059
American Indian or Alaska Native	16 (0.3)	359 (0.3)	375 (0.3)	
Asian	73 (1.2)	1,865 (1.4)	1,938 (1.4)	
Black or African American	1,088 (17.5)	22,598 (17.3)	23,686 (17.3)	
Pacific Islander	3 (0.0)	75 (0.1)	79 (0.1)	
White	4,871 (78.4)	100,969 (77.3)	105,840 (77.4)	
Other/Unknown	163 (2.6)	4,698 (3.6)	4,861 (3.6)	
Length of Stay (Days)				<0.001
Mean (SD)	9.9 (6.0)	4.4 (4.1)	4.4 (4.1)	
Median (IQR)	8.6 (8.7)	3.1 (3.7)	3.1 (3.7)	
^2^Average Number of Pages				<0.001
Mean (SD)	17.1 (16)	12.6 (13.7)	12.8 (13.9)	
Median (IQR)	12 (16)	8 (12)	8 (13)	

^1^P-value of difference in encounters with deterioration event versus encounters without deterioration event

^2^Statistics calculated for cohort experiencing a deterioration event are measured to the time of first event per encounter

**Figure 2:**
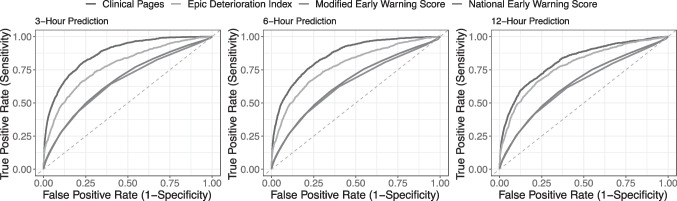
Model discrimination.

The model using clinical pages outperformed the early warning scores on the hold-out testing dataset at each time horizon. Our model to predict deterioration in the next 3-hours yielded the best performance with an AUROC of 0.866 (95% CI [0.865–0.867]) and F-Score of 0.295 (95% CI [0.293–0.297]). We compare discrimination of all four predictive models in Fig. 2. We observed that the Modified Early Warning Score (MEWS) yielded the lowest performance of all models with an AUROC of 0.655 (95% CI [0.655–0.655]) at 3-hours before a deterioration event and an AUROC of 0.635 (95% CI [0.635–0.635]) 12-hours before an event. Classification metrics for all models are available in Table 2. Classification metrics stratified by deterioration event are available in Appendix C.

**Table 2:** Performance of Clinical Page Prediction Model Compared to Commonly Implemented Early Warning Scores

Score, No. (95% CI)						
	AUROC	AUPRC	Sensitivity	Specificity	F-Score	PPV
Clinical Pages						
3-hours	0.866 (0.865–0.867)	0.352 (0.348–0.355)	0.619 (0.615–0.622)	0.890 (0.890–0.891)	0.295 (0.293–0.297)	0.193 (0.192–0.195)
6-hours	0.856 (0.858–0.862)	0.316 (0.311–0.321)	0.590 (0.586–0.595)	0.900 (0.899–0.900)	0.291 (0.289–0.294)	0.193 (0.191–0.195)
12-hours	0.816 (0.814–0.818)	0.249 (0.245–0.252)	0.469 (0.466–0.473)	0.927 (0.926–0.927)	0.275 (0.273–0.277)	0.195 (0.193–0.196)
Epic Deterioration Index^1^						
3-hours	0.781 (0.781–0.782)	0.333 (0.333–0.334)	0.503 (0.502–0.504)	0.878 (0.878–0.879)	0.224 (0.223–0.224)	0.144 (0.143–0.144)
6-hours	0.781 (0.780–0.781)	0.332 (0.331–0.333)	0.505 (0.504–0.506)	0.878 (0.878–0.879)	0.220 (0.220–0.220)	0.141 (0.140–0.141)
12-hours	0.782 (0.782–0.783)	0.330 (0.330–0.331)	0.508 (0.507–0.509)	0.878 (0.878–0.879)	0.212 (0.213–0.214)	0.134 (0.134–0.135)
Modified Early Warning Score (MEWS)						
3-hours	0.655 (0.655, 0.655)	0.275 (0.274–0.275)	0.436 (0.436–0.437)	0.793 (0.792–0.793)	0.147 (0.147–0.147)	0.089 (0.088–0.089)
6-hours	0.640 (0.640–0.641)	0.266 (0.266–0.266)	0.422 (0.422–0.423)	0.793 (0.792–0.793)	0.140 (0.140–0.141)	0.084 (0.084–0.084)
12-hours	0.635 (0.635–0.635)	0.259 (0.259–0.259)	0.416 (0.416–0.417)	0.793 (0.792–0.793)	0.131 (0.131–0.131)	0.078 (0.077–0.078)
National Early Warning Score (NEWS)						
3-hours	0.670 (0.669, 0.670)	0.386 (0.386–0.386)	0.691 (0.691–0.692)	0.555 (0.555–0.555)	0.122 (0.122–0.122)	0.067 (0.067–0.067)
6-hours	0.655 (0.655–0.655)	0.376 (0.375–0.376)	0.673 (0.673–0.674)	0.555 (0.555–0.555)	0.117 (0.117–0.117)	0.064 (0.064–0.064)
12-hours	0.650 (0.650–0.650)	0.370 (0.370–0.370)	0.667 (0.666–0.667)	0.555 (0.555–0.555)	0.109 (0.109–0.109)	0.059 (0.059–0.059)

^1^Epic Deterioration Index data were available beginning July 1, 2020

Using the pre-defined prediction threshold, our model accurately identified 61.9% of deterioration events within 3-hours and 46.9% of events within 12-hours. Table 3 presents results from our sensitivity analysis across prediction thresholds. Within 6-hours, the lowest prediction threshold of 0.1 would accurately identify 74% of patients experiencing a deterioration event within 6-hours with a PPV of 13%. Increasing the PPV to 41% would identify 29% of deterioration events. The best performing early warning score, the Epic Deterioration Index, accurately identified 51% of events within 6-hours with a PPV of 14%.

**Table 3:** Comparison of Classification Metrics by Predicted Probability Cutpoint

	Score, No. (95% CI)			
Cutpoint	Sensitivity	Specificity	F-Score	PPV
3-hours				
0.1	0.747	0.814	0.244	0.146
	(0.744–0.750)	(0.814–0.815)	(0.243–0.246)	(0.145–0.147)
0.2	0.739	0.823	0.250	0.151
	(0.736–0.742)	(0.822–0.823)	(0.249–0.252)	(0.150–0.152)
0.3	0.734	0.827	0.253	0.153
	(0.731–0.737)	(0.827–0.828)	(0.252–0.255)	(0.152–0.154)
0.4	0.730	0.831	0.256	0.155
	(0.727–0.733)	(0.830–0.831)	(0.254–0.258)	(0.154–0.156)
0.5	0.726	0.835	0.259	0.158
	(0.723–0.730)	(0.834–0.835)	(0.257–0.261)	(0.156–0.159)
0.6	0.719	0.841	0.263	0.161
	(0.716–0.722)	(0.840–0.841)	(0.261–0.265)	(0.160–0.162)
0.7	0.710	0.849	0.271	0.167
	(0.707–0.714)	(0.849–0.850)	(0.269–0.273)	(0.166–0.169)
0.8	0.671	0.873	0.288	0.184
	(0.668–0.674)	(0.872–0.873)	(0.286–0.290)	(0.182–0.185)
0.9	0.488	0.948	0.362	0.287
	(0.485–0.491)	(0.948–0.949)	(0.359–0.364)	(0.285–0.290)
6-hours				
0.1	0.738	0.799	0.221	0.130
	(0.735–0.741)	(0.798–0.799)	(0.220–0.223)	(0.129–0.131)
0.2	0.718	0.814	0.228	0.136
	(0.716–0.721)	(0.813–0.814)	(0.227–0.230)	(0.135–0.137)
0.3	0.707	0.823	0.234	0.14
	(0.704–0.710)	(0.822–0.823)	(0.232–0.235)	(0.139–0.141)
0.4	0.696	0.830	0.237	0.143
	(0.693–0.699)	(0.830–0.831)	(0.236–0.239)	(0.142–0.144)
0.5	0.687	0.837	0.242	0.147
	(0.684–0.690)	(0.837–0.838)	(0.240–0.243)	(0.146–0.148)
0.6	0.673	0.846	0.247	0.151
	(0.670–0.676)	(0.846–0.847)	(0.246–0.249)	(0.150–0.153)
0.7	0.649	0.861	0.257	0.16
	(0.646–0.652)	(0.860–0.861)	(0.255–0.258)	(0.159–0.161)
0.8	0.589	0.899	0.290	0.192
	(0.586–0.592)	(0.899–0.899)	(0.288–0.292)	(0.191–0.194)
0.9	0.293	0.983	0.343	0.413
	(0.290–0.296)	(0.983–0.983)	(0.340–0.346)	(0.410–0.417)
12-hours				
0.1	0.548	0.896	0.254	0.166
	(0.543–0.554)	(0.895–0.898)	(0.252–0.256)	(0.164–0.167)
0.2	0.530	0.903	0.258	0.170
	(0.535–0.535)	(0.901–0.905)	(0.256–0.260)	(0.169–0.172)
0.3	0.516	0.909	0.263	0.176
	(0.511–0.521)	(0.908–0.911)	(0.261–0.265)	(0.175–0.178)
0.4	0.494	0.917	0.266	0.183
	(0.488–0.500)	(0.915–0.919)	(0.264–0.269)	(0.181–0.185)
0.5	0.473	0.926	0.274	0.193
	(0.468–0.479)	(0.924–0.927)	(0.272–0.276)	(0.191–0.195)
0.6	0.438	0.938	0.283	0.210
	(0.432–0.444)	(0.936–0.940)	(0.280–0.286)	(0.207–0.212)
0.7	0.376	0.956	0.296	0.246
	(0.369–0.382)	(0.955–0.958)	(0.294–0.299)	(0.243–0.249)
0.8	0.261	0.981	0.295	0.344
	(0.254–0.269)	(0.979–0.982)	(0.292–0.299)	(0.339–0.349)
0.9	0.094	0.998	0.161	0.607
	(0.089–0.098)	(0.997–0.998)	(0.156–0.166)	(0.597–0.617)

## DISCUSSION

We developed a deep learning algorithm to classify imminent clinical deterioration among hospitalized patients. Using text from the sequence of clinical pages sent during routine care, our retrospective analysis found that our models accurately predicted 62% of deterioration events within 3-hours and 47% of deterioration events within 12-hours with good discrimination (AUROC, 0.87–0.82). These results significantly improved upon the best existing, commonly implemented EWS, which yielded AUROCs of 0.781 at 3-hours and 0.782 at 12-hours before a deterioration event.

Clinical pages offer insight into decision making and intuition around key clinical findings. In reviewing pages predicted to demonstrate a high probability of deterioration, we found that messages indicated a mix of expressions of direct concern (i.e., “Please call for critical findings in arterial study”; “Can you come see pt? Significant full body tremors, idk if it’s from anxiety, Valium given 25 mins ago”) and mentions of specific, potentially concerning, findings (i.e., “Pt SBP >140, IV hydralazine given x2, IV pain meds x2”; “BP 90/63 [MAP 72]”). Few studies highlighted the importance of clinical intuition in recognizing clinical deterioration. Romero-Brufau found that a nurse recorded indicator of worry significantly improved the prediction of ICU-transfer in 24-hours.^21^ Nursing worry and clinical concern provides important context that combines both subjective and objective impressions of patient condition.^22, 24, 42^ Douw and colleagues found that descriptions of nurse worry encompass over 170 unique clinical concerns, including impressions that are not easily recorded as objective findings.^24^ Common EWS measure a median of 12 variables.^16^ We hypothesize that our model evaluates a wider array of findings and concerns, which contributes to its improved performance. Pages also provide specific insight into immediate concerns without relying on documentation in the EHR which is often delayed.^43–45^ Nonetheless, it is possible that the combination of clinical pages and structured EHR data may offer improved performance, which we will investigate in future work.

Predicting clinical deterioration must balance adequate time to meaningfully intervene in patient care with a time horizon in which clinically meaningful changes to predictors can be observed. EWS predict clinical deterioration at lengthy time horizons; most commonly exceeding 24-hours.^16^ However, patients who experience deterioration events begin to show signs of clinical instability in the preceding 8 to 12 hours.^46–48^ In comparing predictions using clinical pages with common EWS at the encounter level, we found that performance of our clinical pages algorithm improved closer to the deterioration event, suggesting that pages continue to indicate worrisome trends in the time leading to an event. Common EWS stayed relatively consistent across all time horizons. When evaluating EWS performance only during the time horizon (Appendix B), we note substantially poorer performance that gradually increases with longer time horizons. This reflects prior findings that EWS identify the sickest patients rather than individuals likely to imminently deteriorate.^17, 20^ EWS performance differs by type of event.^5, 49^ Our analysis stratified by event found that predicting cardiac arrest yielded the highest performance, which echoes prior work.^5, 46, 47^ Interestingly, EWS yielded better performance than clinical pages when predicting cardiac arrest – both across the entire encounter and immediately before an event. This suggests that these patients maintain high scores throughout the encounter and that the clinical team may already be aware of the patient’s condition. Our clinical pages model demonstrated marked improvement in predicting ICU transfer or Rapid Responses, suggesting clinical intuition is an important predictor.

Predicting imminent deterioration supports workflows for clinical response and intervention. Hospital quality and safety leaders could incorporate these findings into existing Rapid Response processes by providing a list of high-risk patients to support outreach and rounding support. When trends are detected, urgent messages could communicate findings to the charge nurse and clinical team for assessment and intervention. Highlighting concerning trends can help providers prioritize urgent needs. The algorithm could support automatic calls for Rapid Response support when a patient has a high likelihood of deterioration. Enabling data-driven response to increased patient acuity can improve upon common barriers to calling rapid response based on intuition alone, including feelings of uncertainty.^50, 51^

Using clinical pages to monitor patient acuity integrates key data points without changes to clinical workflow or nursing documentation. Few studies incorporated non-traditional data sources as artifacts of clinical care.^34^ Fu and colleagues measured frequency of documentation in the EHR to predict clinical deterioration in intensive care units with modest performance. Extracting clinical impressions or concerns from clinical documentation has shown promise in some clinical scenarios,^37, 52^ but limitations to timely documentation and frequency of nursing assessments limit utility of these approaches.

Our findings have limitations. We performed this research at a single academic medical center which uses an Epic EHR and relies extensively on pages to communicate between nurses, physicians, and other clinicians. Results may not generalize to other organizations. Our patient population also included a disproportionate number of White patients. These demographic characteristics closely reflect the broad demographics of middle Tennessee but nonetheless introduces potential racial bias in our sample. Future work should seek to better understanding how implicit biases affect clinical paging behavior. While our findings suggest significant improvement in detection of imminent deterioration, this research was conducted as a retrospective analysis. Additional study as a prospective randomized controlled trial should validate the impact of our model to improve clinical care. Our corpus of clinical deterioration events was based on retrospective chart review by a single reviewer. Despite cross-referencing annotated events with data from admission-discharge-transfer feeds, Rapid Response activations, and STAT activations, it is possible that a subset of events may have been incorrectly annotated or some events may have been missed during the annotation process. Finally, it is inconclusive if our findings highlight new clinical concerns versus existing concerns of which the clinical team is already aware. We will test the extent to which our machine learning approach highlights unrecognized instances of clinical urgency or concern in future work.

## CONCLUSION

Our findings suggest that machine learning applied to the content and frequency of clinical pages improves prediction of imminent clinical deterioration. Our models provided improved discrimination at each time interval and outperformed the best performing common early warning scores across all classification metrics. Quantitative clinical measures are integral to patient monitoring but are not a substitute for experience and intuition. Using clinical pages to monitor patient acuity integrates both expert intuition and clinical decision making around key data to improve detection of imminent clinical deterioration without changing clinical workflow or nursing documentation.

### Supplementary Information

ESM 1 (DOCX 13 kb)

ESM 2 (DOCX 15 kb)

ESM 3 (DOCX 19 kb)

ESM 4 (DOCX 17 kb)
