# Supplementary material for: Development and Validation of a Machine Learning Algorithm Using Clinical Pages to Predict Imminent Clinical Deterioration
Source: J Gen Intern Med. 2023 Aug 1;39(1):27–35. doi: 10.1007/s11606-023-08349-3 (PMC10817885; doi:10.1007/s11606-023-08349-3)
Supplement: Supplementary file 1 — (DOCX 13 kb) [file 11606_2023_8349_MOESM1_ESM.docx]

Supplemental Table 1: Hyperparameter Tuning Ranges

| **Parameter** | **Value Set** |
| --- | --- |
| Number of LSTM Layers | {0, 1} |
| LSTM Layer 1 Units | {16 – 512, by 16} |
| LSTM Layer 2 Units | {16 – 512, by 16} |
| Dropout | {0, 0.1, 0.2, 0.3, 0.4, 0.5} |
| Learning Rate | {1e-3, 3e-4, 1e-4, 5e-5, 1e-5} |
