# Supplementary material for: Development and Validation of a Machine Learning Algorithm Using Clinical Pages to Predict Imminent Clinical Deterioration
Source: J Gen Intern Med. 2023 Aug 1;39(1):27–35. doi: 10.1007/s11606-023-08349-3 (PMC10817885; doi:10.1007/s11606-023-08349-3)
Supplement: Supplementary file 2 — (DOCX 15 kb) [file 11606_2023_8349_MOESM2_ESM.docx]

**Appendix B:** Comparison of Early Warning Score Classification Performance During the Time Horizon Immediately Before a Deterioration Event.

If a patient experienced a deterioration event, we obtained the highest EWS score in the k hours before the deterioration event until the deterioration event occurred. If a patient did not experience a deterioration event, we obtained the highest EWS score from the entire hospitalization.

|  | Score, No. (95% CI) | | | | | |
| --- | --- | --- | --- | --- | --- | --- |
|  | **AUROC** | **AUPRC** | **Sensitivity** | **Specificity** | **F-Score** | **PPV** |
| **Epic Deterioration Index^1^** | | | | | | |
| **3-hours** | 0.695  (0.695-0.696) | 0.237  (0.236-0.238) | 0.343  (0.342-0.344) | 0.878  (0.878-0.879) | 0.160  (0.160-0.161) | 0.105  (0.104-0.105) |
| **6-hours** | 0.713  (0.712-0.714) | 0.251  (0.250-0.252) | 0.366  (0.365-0.367) | 0.878  (0.878-0.879) | 0.170  (0.170-0.171) | 0.111  (0.110-0.111) |
| **12-hours** | 0.731  (0.731-0.732) | 0.262  (0.262-0.263) | 0.384  (0.384-0.386) | 0.878  (0.878-0.879) | 0.178  (0.177-0.179) | 0.116  (0.115-0.116) |
| **Modified Early Warning Score (MEWS)** | | | | | | |
| **3-hours** | 0.505  (0.505-0.505) | 0.185  (0.185-0.185) | 0.287  (0.286-0.287) | 0.793  (0.792-0.793) | 0.092  (0.091-0.092) | 0.054  (0.054-0.055) |
| **6-hours** | 0.517  (0.517-0.518) | 0.193  (0.193-0.193) | 0.294  (0.293-0.294) | 0.793  (0.792-0.793) | 0.101  (0.101-0.102) | 0.061  (0.061-0.061) |
| **12-hours** | 0.552  (0.552-0.553) | 0.210  (0.209-0.210) | 0.322  (0.322-0.323) | 0.793  (0.792-0.793) | 0.112  (0.112-0.112) | 0.068  (0.068-0.068) |
| **National Early Warning Score (NEWS)** | | | | | | |
| **3-hours** | 0.542  (0.542-0.542) | 0.286  (0.285-0.286) | 0.507  (0.506-0.507) | 0.555  (0.555-0.555) | 0.083  (0.083-0.083) | 0.045  (0.045-0.045) |
| **6-hours** | 0.555  (0.555-0.555) | 0.300  (0.299-0.300) | 0.527  (0.526-0.527) | 0.555  (0.555-0.555) | 0.094  (0.094-0.095) | 0.052  (0.052-0.052) |
| **12-hours** | 0.586  (0.586-0.587) | 0.324  (0.324-0.324) | 0.572  (0.571-0.572) | 0.555  (0.555-0.555) | 0.103  (0.103-0.104) | 0.057  (0.057-0.057) |
