# Supplementary material for: Development and Validation of a Machine Learning Algorithm Using Clinical Pages to Predict Imminent Clinical Deterioration
Source: J Gen Intern Med. 2023 Aug 1;39(1):27–35. doi: 10.1007/s11606-023-08349-3 (PMC10817885; doi:10.1007/s11606-023-08349-3)
Supplement: Supplementary file 3 — (DOCX 19 kb) [file 11606_2023_8349_MOESM3_ESM.docx]

**Appendix C:** Comparison of Clinical Page Prediction Model Classification Performance by Type of Deterioration Event. AUC, Sensitivity, and Specificity are classification measures invariant of case prevalence; PPV, F-Score, and AUPRC are highly affected by prevalence.

|  | Score, No. (95% CI) | | | | | |
| --- | --- | --- | --- | --- | --- | --- |
|  | **AUROC** | **AUPRC** | **Sensitivity** | **Specificity** | **F-Score** | **PPV** |
| **Clinical Pages** | | | | | | |
| **In-Hospital Cardiac Arrest** | | | | | | |
| **3-hours** | 0.919  (0.914, 0.923) | 0.120  (0.111, 0.129) | 0.712  (0.696, 0.728) | 0.908  (0.906, 0.911) | 0.037  (0.036, 0.039) | 0.019  (0.018, 0.020) |
| **6-hours** | 0.898  (0.892, 0.904) | 0.156  (0.145, 0.167) | 0.692  (0.677, 0.708) | 0.899  (0.899, 0.900) | 0.032  (0.031, 0.034) | 0.017  (0.016, 0.017) |
| **12-hours** | 0.894  (0.890, 0.900) | 0.131  (0.122, 0.140) | 0.615  (0.601, 0.630) | 0.932  (0.931, 0.932) | 0.042  (0.041, 0.044) | 0.020  (0.020, 0.021) |
| **Rapid Response Activation or ICU Transfer** | | | | | | |
| **3-hours** | 0.869  (0.868, 0.870) | 0.318  (0.314, 0.322) | 0.681  (0.678, 0.684) | 0.857  (0.857, 0.858) | 0.260  (0.258, 0.262) | 0.161  (0.159, 0.162) |
| **6-hours** | 0.856  (0.855, 0.858) | 0.292  (0.288, 0.296) | 0.659  (0.656, 0.662) | 0.853  (0.852, 0.853) | 0.239  (0.238, 0.241) | 0.146  (0.145, 0.147) |
| **12-hours** | 0.812  (0.810, 0.814) | 0.225  (0.222, 0.229) | 0.446  (0.442, 0.449) | 0.932  (0.932, 0.933) | 0.264  (0.262, 0.267) | 0.188  (0.186, 0.190) |
| **Epic Deterioration Index^1^** | | | | | | |
| **In-Hospital Cardiac Arrest** | | | | | | |
| **3-hours** | 0.979  (0.978, 0.979) | 0.513  (0.513, 0.514) | 0.963  (0.962, 0.964) | 0.878  (0.878, 0.879) | 0.119  (0.118, 0.119) | 0.063  (0.063, 0.063) |
| **6-hours** | 0.975  (0.974, 0.975) | 0.510  (0.510, 0.511) | 0.958  (0.957, 0.959) | 0.878  (0.878, 0.879) | 0.117  (0.117, 0.118) | 0.062  (0.062, 0.063) |
| **12-hours** | 0.970  (0.970, 0.971) | 0.506  (0.505, 0.506) | 0.950  (0.950, 0.951) | 0.878  (0.878, 0.879) | 0.116  (0.115, 0.116) | 0.062  (0.061, 0.062) |
| **Rapid Response Activation or ICU Transfer** | | | | | | |
| **3-hours** | 0.729  (0.728, 0.729) | 0.245  (0.245, 0.246) | 0.381  (0.379, 0.382) | 0.878  (0.878, 0.879) | 0.147  (0.147, 0.148) | 0.091  (0.091, 0.092) |
| **6-hours** | 0.728  (0.728, 0.729) | 0.244  (0.244, 0.245) | 0.382  (0.381, 0.383) | 0.878  (0.878, 0.879) | 0.144  (0.143, 0.144) | 0.089  (0.089, 0.089) |
| **12-hours** | 0.727  (0.726, 0.727) | 0.239  (0.238, 0.239) | 0.379  (0.377, 0.379) | 0.878  (0.878, 0.879) | 0.135  (0.134, 0.135) | 0.082  (0.082, 0.082) |
| **Modified Early Warning Score** | | | | | | |
| **In-Hospital Cardiac Arrest** | | | | | | |
| **3-hours** | 0.925  (0.925, 0.926) | 0.461  (0.460, 0.462) | 0.908  (0.907, 0.909) | 0.793  (0.793, 0.793) | 0.027  (0.027, 0.027) | 0.014  (0.014, 0.014) |
| **6-hours** | 0.914  (0.914, 0.915) | 0.448  (0.448 0.449) | 0.883  (0.882, 0.885) | 0.793  (0.793, 0.793) | 0.026  (0.026, 0.026) | 0.013  (0.013, 0.013) |
| **12-hours** | 0.901  (0.901, 0.902) | 0.440  (0.439, 0.441) | 0.867  (0.865, 0.869) | 0.793  (0.793, 0.793) | 0.025  (0.025, 0.025) | 0.013  (0.013, 0.013) |
| **Rapid Response Activation or ICU Transfer** | | | | | | |
| **3-hours** | 0.635  (0.635, 0.636) | 0.252  (0.251, 0.252) | 0.402  (0.401, 0.402) | 0.793  (0.793, 0.793) | 0.129  (0.128, 0.129) | 0.077  (0.077, 0.077) |
| **6-hours** | 0.620  (0.619, 0.620) | 0.242  (0.242, 0.243) | 0.388  (0.387, 0.388) | 0.793  (0.793, 0.793) | 0.122  (0.122, 0.123) | 0.073  (0.072, 0.073) |
| **12-hours** | 0.614  (0.613, 0.614) | 0.235  (0.235, 0.236) | 0.381  (0.380, 0.381) | 0.793  (0.793, 0.793) | 0.113  (0.113, 0.114) | 0.067  (0.067, 0.067) |
| **National Early Warning Score** | | | | | | |
| **In-Hospital Cardiac Arrest** | | | | | | |
| **3-hours** | 0.935  (0.935, 0.936) | 0.493  (0.492, 0.493) | 0.978  (0.977, 0.978) | 0.555  (0.555, 0.556) | 0.014  (0.014, 0.014) | 0.007  (0.007, 0.007) |
| **6-hours** | 0.925  (0.924, 0.926) | 0.490  (0.489, 0.490) | 0.973  (0.972, 0.973) | 0.555  (0.555, 0.556) | 0.014  (0.014, 0.014) | 0.007  (0.007, 0.007) |
| **12-hours** | 0.916  (0.916, 0.917) | 0.490  (0.489, 0.490) | 0.972  (0.972, 0.973) | 0.555  (0.555, 0.556) | 0.013  (0.013, 0.013) | 0.007  (0.007, 0.007) |
| **Rapid Response Activation or ICU Transfer** | | | | | | |
| **3-hours** | 0.650  (0.650, 0.650) | 0.372  (0.372, 0.373) | 0.670  (0.670, 0.671) | 0.555  (0.555, 0.556) | 0.112  (0.111, 0.112) | 0.061  (0.061, 0.061) |
| **6-hours** | 0.635  (0.634, 0.635) | 0.361  (0.361, 0.362) | 0.651  (0.650, 0.651) | 0.555  (0.555, 0.556) | 0.106  (0.106, 0.106) | 0.058  (0.058, 0.058) |
| **12-hours** | 0.629  (0.629, 0.630) | 0.629  (0.630, 0.630) | 0.643  (0.644, 0.645) | 0.555  (0.555, 0.556) | 0.099  (0.099, 0.099) | 0.053  (0.053, 0.054) |

^1^Epic Deterioration Index data were available beginning July 1, 2020.
