# Supplementary material for: Development and Validation of a Machine Learning Algorithm Using Clinical Pages to Predict Imminent Clinical Deterioration
Source: J Gen Intern Med. 2023 Aug 1;39(1):27–35. doi: 10.1007/s11606-023-08349-3 (PMC10817885; doi:10.1007/s11606-023-08349-3)
Supplement: Supplementary file 4 — (DOCX 17 kb) [file 11606_2023_8349_MOESM4_ESM.docx]

**Appendix D:** Comparison of Early Warning Score Classification Performance, by Type of Deterioration Event, During the Time Horizon Immediately Before the Event.

If a patient experienced a deterioration event, we obtained the highest EWS score in the k hours before the deterioration event until the deterioration event occurred. If a patient did not experience a deterioration event, we obtained the highest EWS score from the entire hospitalization.

|  | Score, No. (95% CI) | | | | | |
| --- | --- | --- | --- | --- | --- | --- |
|  | **AUROC** | **AUPRC** | **Sensitivity** | **Specificity** | **F-Score** | **PPV** |
| **Epic Deterioration Index^1^** | | | | | | |
| **In-Hospital Cardiac Arrest** | | | | | | |
| **3-hours** | 0.968  (0.968, 0.968) | 0.493  (0.493, 0.495) | 0.925  (0.925, 0.928) | 0.878  (0.878, 0.879) | 0.114  (0.114, 0.115) | 0.061  (0.061, 0.061) |
| **6-hours** | 0.971  (0.971, 0.972) | 0.495  (0.495, 0.496) | 0.929  (0.928, 0.930) | 0.878  (0.878, 0.879) | 0.115  (0.114, 0.115) | 0.061  (0.061, 0.061) |
| **12-hours** | 0.973  (0.972, 0.973) | 0.498  (0.497, 0.499) | 0.933  (0.933, 0.935) | 0.878  (0.878, 0.879) | 0.115  (0.115, 0.116) | 0.061  (0.061, 0.062) |
| **Rapid Response Activation or ICU Transfer** | | | | | | |
| **3-hours** | 0.625  (0.624, 0.625) | 0.134  (0.133, 0.134) | 0.193  (0.192, 0.193) | 0.878  (0.878, 0.879) | 0.079  (0.078, 0.079) | 0.050  (0.049, 0.050) |
| **6-hours** | 0.647  (0.645, 0.647) | 0.151  (0.150, 0.152) | 0.221  (0.220, 0.222) | 0.878  (0.878, 0.879) | 0.090  (0.089, 0.090) | 0.056  (0.056, 0.057) |
| **12-hours** | 0.669  (0.668, 0.670) | 0.164  (0.163, 0.165) | 0.242  (0.241, 0.243) | 0.878  (0.878, 0.879) | 0.098  (0.098, 0.099) | 0.062  (0.061, 0.062) |
| **Modified Early Warning Score** | | | | | | |
| **In-Hospital Cardiac Arrest** | | | | | | |
| **3-hours** | 0.841  (0.840, 0.842) | 0.381  (0.379, 0.382) | 0.752  (0.750, 0.754) | 0.793  (0.793, 0.793) | 0.017  (0.017, 0.017) | 0.009  (0.008, 0.009) |
| **6-hours** | 0.836  (0.835, 0.837) | 0.371  (0.371, 0.373) | 0.732  (0.730, 0.735) | 0.793  (0.793, 0.793) | 0.020  (0.020, 0.020) | 0.010  (0.010, 0.010) |
| **12-hours** | 0.858  (0.857, 0.859) | 0.394  (0.393, 0.394) | 0.775  (0.773, 0.777) | 0.793  (0.793, 0.793) | 0.023  (0.023, 0.023) | 0.012  (0.011, 0.012) |
| **Rapid Response Activation or ICU Transfer** | | | | | | |
| **3-hours** | 0.485  (0.485, 0.485) | 0.167  (0.167, 0.167) | 0.259  (0.259, 0.260) | 0.793  (0.793, 0.793) | 0.079  (0.079, 0.079) | 0.047  (0.047, 0.047) |
| **6-hours** | 0.496  (0.496, 0.497) | 0.174  (0.173, 0.174) | 0.265  (0.264, 0.265) | 0.793  (0.793, 0.793) | 0.087  (0.087, 0.087) | 0.052  (0.052, 0.052) |
| **12-hours** | 0.531  (0.530, 0.531) | 0.189  0.189, 0.189) | 0.290  (0.290, 0.291) | 0.793  (0.793, 0.793) | 0.096  (0.096, 0.096) | 0.058  (0.58, 0.058) |
| **National Early Warning Score** | | | | | | |
| **In-Hospital Cardiac Arrest** | | | | | | |
| **3-hours** | 0.871  (0.871, 0.872) | 0.472  (0.472, 0.473) | 0.939  (0.938, 0.940) | 0.555  (0.555, 0.556) | 0.010  (0.010, 0.010) | 0.005  (0.005, 0.005) |
| **6-hours** | 0.875  (0.874, 0.876) | 0.471  (0.471, 0.472) | 0.937  (0.935, 0.937) | 0.555  (0.555, 0.556) | 0.012  (0.012, 0.012) | 0.006  (0.006, 0.006) |
| **12-hours** | 0.888  (0.888, 0.889) | 0.478  (0.477, 0.478) | 0.948  (0.947, 0.949) | 0.555  (0.555, 0.556) | 0.013  (0.013, 0.013) | 0.007  (0.007, 0.007) |
| **Rapid Response Activation or ICU Transfer** | | | | | | |
| **3-hours** | 0.522  (0.521, 0.522) | 0.270  (0.270, 0.271) | 0.480  (0.480, 0.481) | 0.555  (0.555, 0.555) | 0.075  (0.075, 0.075) | 0.041  (0.041, 0.041) |
| **6-hours** | 0.534  (0.533, 0.534) | 0.283  (0.283, 0.283) | 0.499  (0.499, 0.500) | 0.555  (0.555, 0.555) | 0.085  (0.084, 0.085) | 0.046  (0.046, 0.046) |
| **12-hours** | 0.565  (0.564, 0.565) | 0.307  (0.307, 0.307) | 0.545  (0.544, 0.545) | 0.555  (0.555, 0.555) | 0.093  (0.093, 0.093) | 0.051  (0.051, 0.051) |

^1^Epic Deterioration Index data were available beginning July 1, 2020.
